# Integrating intimate partner violence prevention content into a digital parenting chatbot intervention during COVID-19: Intervention development and remote data collection

**DOI:** 10.1186/s12889-023-16649-w

**Published:** 2023-09-04

**Authors:** Moa Schafer, Jamie M. Lachman, Frances Gardner, Paula Zinser, Francisco Calderon, Qing Han, Chiara Facciola, Lily Clements

**Affiliations:** 1 Centre for Evidence Based Intervention, Department of Social Policy and Intervention, University of Oxford, Oxford, United Kingdom; 2 Social and Public Health Sciences Unit, University of Glasgow, Glasgow, Scotland; 3 Centre for Social Science Research, University of Cape Town, Cape Town, South Africa; 4 IDEMS International, Berkshire, United Kingdom

**Keywords:** Intimate partner violence, Violence against children, COVID-19, Parenting, Intervention development

## Abstract

**Background:**

Intimate partner violence (IPV) is a serious public health issue which experienced a sharp incline during the onset of COVID-19. Increases in other forms of violence, such as violence against children (VAC), have also been linked to the pandemic, and there have been calls for greater prevention efforts that tackle both forms of violence concurrently. The COVID-19 crisis has highlighted the urgent need for evidence-based and scalable violence prevention interventions that target multiple forms of family violence. Parenting programmes have shown promising results in preventing various forms of family violence, including IPV and VAC, and have recently experienced an expansion in delivery, with digital intervention formats growing. This paper describes the development and evaluation of the IPV prevention content designed and integrated into ParentText, a chatbot parenting intervention adapted from Parenting for Lifelong Health programmes.

**Methods:**

The ParentText IPV prevention content was developed using the Six Steps in Quality Intervention Development (6SQuID) framework. This involved targeted literature searches for key studies to identify causal factors associated with IPV and determining those with greatest scope for change. Findings were used to develop the intervention content and theory of change. Consultations were held with academic researchers (*n* = 5), practitioners (*n* = 5), and local community organisations (*n* = 7), who reviewed the content. A formative evaluation was conducted with parents in relationships (*n* = 96) in Jamaica to better understand patterns in user engagement with the intervention and identify strategies to further improve engagement.

**Results:**

Using the 6SQuID model, five topics on IPV prevention were integrated into the ParentText chatbot. Text-messages covering each topic, including additional materials such as cartoons and videos, were also developed. The formative evaluation revealed an average user-engagement length of 14 days, 0.50 chatbot interactions per day, and over half of participants selected to view additional relationship content.

**Conclusions:**

This article provides a unique contribution as the first to integrate IPV prevention content into a remotely delivered, digital parenting intervention for low-resource settings. The findings from this research and formative evaluation shed light on the promising potential of chatbots as scalable and accessible forms of violence prevention, targeting multiple types of family violence.

**Supplementary Information:**

The online version contains supplementary material available at 10.1186/s12889-023-16649-w.

## Background

Intimate partner violence (IPV) is a serious and pervasive issue, which has both acute and long-standing effects on women and families worldwide [1]. IPV is associated with a range of short- and long-term physical and mental health consequences including physical injuries, mental health issues, chronic disease, and stress (WHO, 2012). IPV is the most common form of violence experienced by women [2] and global estimates indicate that, in their lifetime, 1 in 3 women experience IPV [3]. The onset of the COVID-19 pandemic saw rates of IPV, including other forms of violence in the home such as violence against children, sharply increase [4]. Notably, the co-occurrence of IPV and other forms of violence in the household has received growing attention in recent years [5], with a particular focus on the intersections that exist between IPV and violence against children [6]. In the past, efforts to address these two forms of violence have developed predominantly in separate trajectories [6]. However, increasingly, global calls have been made for more integrated forms of prevention to urgently tackle both types of violence concurrently [7]. The surge in rates of both IPV and violence against children following the arrival of COVID-19, have further underscored this pressing need for integrated and scalable violence prevention efforts [4].

Parenting interventions have been identified as an effective strategy to address and prevent multiple forms of family violence, including IPV and violence against children [6]. For example, a randomized controlled trial (RCT) of a group-based parenting programme in the Philippines found significant reductions in both child maltreatment and also in IPV at post-test [8]. In terms of preventing violence against children, parenting programmes have shown a number of promising results [9, 10]. Findings from a meta-analysis by Chen and Chan [11], for example, found that parenting programmes prevented child maltreatment by enhancing protective factors and reducing risk factors. Likewise, a systematic review for the 2023 WHO Guideline on Parenting Interventions found 49 randomized trials showing consistent beneficial effects of parenting interventions for reducing maltreatment and harsh parenting [9]. In a similar vein, fatherhood interventions, which focus on the involvement of and engagement between fathers and their children, are also gaining recognition in the violence prevention literature [12]. Whilst parenting interventions have often faced challenges with recruiting and engaging fathers due to cultural and logistical barriers [13], in recent years there have been an increase in interventions that focus specifically on fathers and improving their engagement [14]. Indeed, interventions that involve men and boys have been identified as a unique and valuable opportunity to address masculine norms that contribute to child maltreatment and gender-based violence [15], through activities that promote new behaviours and self-reflection encouraging positive involvement as fathers [16].

Globally, interventions that involve men to prevent the perpetration of IPV are gradually becoming more common [17]. The community mobilisation intervention SASA! in Uganda, for example, was designed to change community norms, attitudes, and behaviours that lead to gender inequality and violence [18]. Results from a pair-matched cluster RCT of SASA! found a number of promising findings. For instance, compared to their control counterparts, women in intervention communities were less likely to have experienced all types of IPV (physical, sexual, emotional, and controlling behaviours) at follow-up [19].

While there is extensive evidence of the effectiveness of parenting programmes across various well-being and psychosocial outcomes, the reach of parenting programmes is often limited due to the accessibility and reach of the interventions [20]. Structural barriers in LMICs, such as high costs, limited infrastructure, and human resources, often create challenges in programme delivery and implementation [21, 22]. There is also a need for programme implementation on a larger scale to achieve greater reach and programme impact and reflect the widespread incidence of VAC and IPV on population levels [23]. Responding to this need, there has been a substantial increase over the past two decades in the use of digital technology for the delivery of interventions [24, 25]. Indeed, mobile technology is increasingly being used to deliver interventions, particularly in LMICs, to overcome barriers with access and reach [26, 27]. Digital modalities, such as apps, video conferencing, and chatbots, thus provide promising possibilities in terms of overcoming challenges associated with reach and limited resources [28]. The emergence of COVID-19 further underscored the importance of digital and remote delivery of interventions [29]. Digital modalities thus provide promising possibilities in terms of overcoming challenges associated with reach and limited resources [28].

The flexible format of digitally delivered interventions has also been suggested to make participation easier and increase participation rates, especially for populations that previously may not have attended programmes such as male caregivers and working parents, and the format has also been identified as potentially more cost-effective [28]. Digital programmes also offer unique opportunities for intervention content to be personalised, a strategy which in existing research on text messaging-based interventions has found to increase intervention efficacy [30], as well as provide users with more targeted and relevant material [31]. In recent years, there has been a rise in emerging evidence underscoring the effectiveness of digital and text messaging-based interventions shown to improve various health and wellbeing outcomes through the use of intervention content delivered via text [32]. There also exist numerous studies demonstrating that text-based messaging interventions can modify parental behaviour by, for example, breaking down behaviour change activities into individual steps, helping to refocus parents’ attention, and providing personalized messages that remind parents to more frequently engage in the desired behaviour [33, 34]. Despite the increase in digital interventions and in programmes addressing multiple forms of violence, there still remains a paucity of empirical evidence on digital prevention efforts that seek to address both forms of violence [35]. This gap in the literature thus underscores the need for further research and programmatic efforts in this area.

### Parenting for lifelong health and digital innovations

In response to COVID-19, a series of digital adaptations of the in-person Parenting for Lifelong Health (PLH) programmes were produced, including the development of ParentText, a chatbot intervention for parents and caregivers of children aged 0 to 17 years. ParentText sends automated messages to users via social messaging platforms such as WhatsApp, Telegram, and Facebook messenger, and is also available via SMS for individuals without smartphone access. The parenting content of ParentText was derived from the in-person PLH programmes [36, 37] and has been locally adapted collaboratively with implementing partners in various low- and middle-income countries (LMICs), including in South Africa, Jamaica, the Philippines, Malaysia, and Sri Lanka, to make sure the content is culturally relevant. The material has also been translated into the local languages of the implementing countries. Enrolled participants receive ParentText messages over five weeks. The specific content the user receives in ParentText aligns with the children’s development stage (0–23 months, 2–9 years, 10–17 years) and is delivered via a range of formats including text messages, images, audio, and video for parents and caregivers. Users receive parenting content on the three main themes: 1) relationship building by spending time together, 2) positive reinforcement, 3) stress reduction for parents and caregivers. ParentText also includes material on additional topics including child development, children living with disabilities, online child safety, talking about COVID-19 with children, family budgeting, helping with schoolwork, family harmony, sexual violence prevention, and partner relationships – the latter of which is the primary focus of this research project (ClinicalTrials NCT05003518). In addition, participants receive troubleshooting messages corresponding to the material to see whether they practised the skill, how it went, and to identify what challenges they may have experienced.

Using the Six Steps in Quality Intervention Development (6SQuID) framework [38], this paper sets out to develop, create, and evaluate IPV prevention content embedded in the digital intervention ParentText. More specifically, this article seeks to answer the following research question regarding the feasibility of the IPV prevention content in ParentText: What is the retention rate and level of user engagement with IPV prevention content developed and integrated into the digital intervention ParentText for parents and caregivers above 16 years of age? Accordingly, the present paper is organised into three sections: develop (Steps 1–2), create (Steps 3–4), and evaluate (Step 5), with the first two sections (Steps 1–4) discussing the empirical development process and the creation of the intervention content, and the third section (Step 5) focusing on the formative evaluation examining the retention rate and user engagement of the developed IPV prevention content.

## Methods

### Study approach

This study reports on the formative research carried out to develop, create, and evaluate the IPV prevention content and assessments integrated in the ParentText programme and follows the Six Steps in Quality Intervention Development (6SQuID) model [38]. The 6SQuID framework builds upon existing intervention development models, such as the UK Medical Research Council’s (MRC) guidance for developing and evaluating complex interventions [39, 40], by offering a detailed series of practical steps (see Table 1). The 6SQuID model has been used in various contexts to design and develop behaviour change interventions, including gender-based violence prevention [41] and digital parent support programmes [42].

**Table 1:** Summary of the 6SQuID intervention development framework [38]

6SQuID Steps	Application in the present study
**Step 1:** Define and understand the problem	• Conduct targeted literature search to identify risk factors associated with IPV • Review existing literature to clarify intersections between IPV and other forms of family violence (e.g., violence against children) • Examine evidence base on the intergenerational effects of IPV
**Step 2:** Clarify which causal or contextual factors are malleable and have greatest scope for change	• Carry out targeted review of existing literature on IPV prevention and theoretical frameworks • Conduct a targeted literature search to identify which risk factors and social norms are addressed in existing IPV interventions
**Step 3:** Identify how to bring about change: the change mechanism	• Use findings from targeted literature searches to identify patterns and type of content included in interventions with promising findings • Develop a Theory of Change and IPV prevention and assessment materials using empirical findings from Steps 1–3 • Conduct consultations with grassroot organisation partners (*n* = 7), academic researchers (*n* = 5), and practitioners (*n* = 5) to test intervention content, revise materials, and carry out surface level adaptations and translations
**Step 4:** Identify how to deliver the change mechanism	• Determine content structure, frequency, and length to develop an intervention content schedule • Hold virtual consultation sessions with grassroots organisation partners (*n* = 7) and academic researcher stakeholder group (*n* = 5) to receive feedback and make amendments on programme structure
**Step 5:** Test and refine on a small scale	• Conduct a small scale formative evaluation (*n* = 96) in selected Kingston Jamaica to test user engagement with the intervention content, with participants recruited through UNICEF Jamaica • Measure and analyse user engagement outcomes to examine interactions with the chatbot • Evaluate findings and identify areas for further improvement and refinement
**Step 6:** Collect sufficient evidence of effectiveness to justify rigorous implementation or evaluation	• Pre-post user-testing study in Jamacia and South Africa currently underway to test preliminary effectiveness, which will be reported in a subsequent forthcoming paper

*6SQUID *Six Steps in Quality Intervention Development Framework

### Study setting

The intervention development was carried out globally in consultation with researchers and practitioners in five LMICs (South Africa, Jamaica, Malaysia, the Philippines, and Sri Lanka) to account for variations in contexts in preparation for scaling up the intervention in the future. These countries were purposefully selected because of pre-existing partnerships with research institutions and organisations in the locations, and because of the high rates of IPV reported in these countries [43–47].The formative evaluation was carried out in Kingston, Jamaica in order to assess engagement with the chatbot and the intervention content. Jamaica was selected as the location for the formative evaluation due to the high levels of IPV reported in the country and because existing collaborations with research partners and community organisations in the country made rollout more feasible.

### Procedures

The development of the ParentText IPV prevention content and assessment was guided by the first five steps of SQuID model: 1) define and understand the problem and its underlying causes; 2) identify which contextual or causal factors have greatest scope for change; 3) clarify how to bring about change; 4) select how to deliver the mechanisms of change; 5) test and adapt on a small scale [38]. The sixth step, 6) ‘Collect sufficient evidence of effectiveness to justify rigorous implementation or evaluation’ (35: p.521), is being pursued through a pre-post user-testing study that is currently being conducted in South Africa and Jamaica, which will be reported in a separate forthcoming paper. A summary of each step is provided in Table 1. A variety of research strategies were adopted during the intervention development and evaluation. In the Results section, an overview of the methods adopted for each phase is provided. For clarity, a summarised overview of the data collection and analysis process of each step is provided below.

### Step 1. ‘Define and understand the problem’

#### Data collection and analysis

In Step 1, we conducted targeted literature searches to review published research and consulted leading researchers in the field of violence prevention to gain a better understanding of risk factors associated with intimate partner violence (IPV) and major points of intersections it shares with other forms of violence in the family, such as violence against children (VAC). The pragmatic approach adopted consisted of a combination of identifying applicable systematic reviews to begin with, hand-searching relevant studies in systematic reviews, and conducting a targeted literature search for applicable papers. Relevant literature was searched for on various academic databases, including, Cochrane Database of Systematic Reviews, PsycINFO, and Pubmed, as well as on websites and databases with unpublished studies, such as Refworld. Key search terms related to risk factors associated with IPV and other forms of family violence were used. Articles recommended by experts were also reviewed. Articles and studies identified were reviewed and analysed in NVivo (version 12) software, which allows for full-text reviewing, annotating, and coding.

### Step 2. ‘Clarify which causal or contextual factors are malleable and have greatest scope for change’

#### Data collection and analysis

For Step 2 we sought to identify which underlying contextual and causal factors associated with IPV have the greatest potential for change. For this step, we built on our empirical findings from Step 1, and extended our review to examine the content and results of existing evidence-based interventions seeking to prevent IPV or address IPV risk factors. Articles identified from this extended review were imported and analysed in NVivo. Casual and contextual factors highlighted in these studies, as well as notable active ingredients identified in the intervention articles, were coded in NVivo and extrapolated as key findings and as factors with notable scope for change. Research results which demonstrated significant intervention effects in relation to reductions in IPV or associated risk factors, such as attitudes condoning IPV, were used to guide the reviewing process.

### Step 3. ‘Identify how to bring about change: the change mechanism’

#### Data collection and analysis

Step 3 focuses on identifying what the change mechanism is, in other words, understanding what process triggers and achieves the desired change in the intervention [38]. For this, we examined and analysed how change could be achieved to the modifiable risk factors identified in Step 2. We developed a theory of change to map out how to bring about the change and developed intervention content in various modalities (text, image, video, and audio) that would allow us to deliver the change mechanism in the identified factors. The content was tested, analysed, and reviewed by grassroots organisation partners (*n* = 7) in virtual consultation sessions and reviewed by academic researchers (*n* = 5) and practitioners (*n* = 5), who provided written and verbal input via email and in individual consultation meetings (see Additional file 5. Consultation focal points for full list of the organisations and practitioners). The analysis process during the consultations included reviewing the content both in terms of theory, cultural sensitivity, and feasibility. The intervention content also underwent translations and surface-level adaptations, which involves changes that are made to intervention material to make sure it matches characteristics of the target populations (e.g. language, music, illustrations) [48].

#### Participants

The grassroot organisations (*n* = 7), researchers (*n* = 5), and practitioners (*n* = 5) who were consulted to review the intervention content were recruited via purposive and convenience sampling from a variety of potential user countries. These organisations and individuals were identified from a network of partner institutions and initiatives with whom the research team had existing relationships and selected based on their work and expertise in the field of family interventions and violence prevention (see Additional file 5 for further details).

### Step 4. ‘Identify how to deliver the change mechanism’

#### Data collection and analysis

Step 4 in 6SQuID is based on determining how to *deliver* the change mechanism [38]. Here we drew upon the risk factors identified in the review and analysis carried out in Step 1 and 2, and the theory of change and content we developed in Step 3, to create a programme schedule. This involved determining what structure, frequency, and length to use, and consulting the grassroots organisation partners (*n* = 7) and the researcher stakeholder group (*n* = 5) (listed in Additional file 5) to receive feedback and make amendments based on their analysis of the proposed structure.

### Step 5. ‘Test and refine on a small scale’

#### Data collection and analysis

Step 5 focuses on examining the feasibility of the intervention content and delivery, as such, we conducted a formative evaluation to test the chatbot and further refine the intervention material. For this, partnered parents and caregivers (*n* = 96) were recruited in Kingston, Jamaica, through convenience sampling carried out through UNICEF Jamaica to test the ParentText chatbot (described in more detail in the Results section). Recruited parents signed up to the chatbot by texting the word ‘PARENT’ to a designated sign-up number on WhatsApp, which then enrolled them in the programme. The aim of this formative evaluation was to help us better understand user engagement with the chatbot and how the intervention could be improved.

### Step 6. ‘Collect sufficient evidence of effectiveness to justify rigorous implementation or evaluation’

This paper focuses on steps 1–5 of the 6SQuID model, and the procedures and results of Step 6 are beyond the scope of this article and will be reported in a separate, forthcoming paper.

## Results

### Step 1: Define and understand the problem

The targeted literature searches conducted in Step 1 sought to understand the risk factors associated with intimate partner violence (IPV) as well as intersections, potential mechanisms of change and risk factors that IPV shares with other forms of violence in the family, such as violence against children. We synthesised the findings from Step 1 by focusing on three main intersections identified between IPV and child maltreatment that have been highlighted in the literature (see Table 2) [6]. These intersections help us to understand the risk factors associated with IPV in relation to other forms of violence, thus identifying areas of overlap which may serve as key entry points and mechanisms of change for integrated violence prevention strategies. The three major intersections identified between IPV and child maltreatment (CM) are outlined below and include: 1) common risk factors and violence co-occurrence; 2) similar consequences and inter-generational effects; and 3) shared social norms [6].

**Table 2:** Summary of major intersections between IPV and violence against children [6]

Intersections between IPV and violence against children
• **Common risk factors and violence co-occurrence**
• **Similar consequences and inter-generational effects**
• **Shared social norms**

### Common risk factors and violence co-occurrence

A major intersection between IPV and CM includes the risk factors they share [6], including society- and family-level risk factors (e.g., poverty and acceptability of family violence) and perpetrator-level risk factors (e.g., parental history of physical abuse, poor mental health, and substance abuse) [49, 50]. This intersection has been gaining attention as scholars start to explore ways of addressing shared risk factors between IPV and VAC as an avenue of addressing shared mechanisms of change in attempts to reduce both forms of violence concurrently [51]. Social and gender norms that condone violence are also a notable shared risk factor [6], as discussed in greater detail below. Another critical intersection is violence co-occurrence, that is, the occurrence of both forms of violence in the same home during the same time period [52].

### Similar consequences and inter-generational effects

The similar consequences shared by IPV and CM are also notable [52], both have impacts that are severe and long-term. Both are linked with serious negative effects on mental and physical health [53, 54]. IPV and CM also share intergenerational consequences. IPV has been found to lead to an increase in pregnancy-associated mental health problems, such as postpartum depression, which negatively impacts a mother’s ability to provide sensitive care for her children [55]. In terms of child maltreatment, studies have found that men who experience physical abuse as children are at greater risk of perpetrating IPV as adults [56, 57]. Studies also suggest that IPV may be a causal factor for child maltreatment [58]. A study by Chan et al. [58], for example, found that IPV during pregnancy was a factor predicting physical abuse against children [58].

### Shared social norms

Another important intersection are social norms, defined as ‘rules of action shared by people in a given society or group; they define what is considered normal and acceptable behaviour for the members of that group’ ([59]: p.409). Gender norms are a subtype of social norms that ‘define acceptable and appropriate actions for women and men in a given group or society’ ([59]: p.415). Scholars suggest that social norms play a key role in not only sustaining VAC but also IPV [51]. Wessells and Kostelny [60] note how social norms often underpin the use of corporal punishment. In some contexts, for example, parents may believe that all parents use harsh parenting in response to unruly child behaviour, and that other parents would criticise them if they did not beat their children for being disobedient [60]. Various social norms have also been linked to a women’s risk of experiencing IPV, including men’s dominance over women, men’s acceptance of wife-beating, and the use of violence to resolve conflict [61]. Indeed, research studies have found that patriarchal gender norms are frequently related to both the occurrence of violence against women and violence against children [62]. Given the growing recognition of these overlapping harmful social norms, there is an increasing consensus on the importance of utilising prevention efforts that address both forms of violence simultaneously [51].

### Step 2: Clarify which causal or contextual factors are malleable

For Step 2, a mixture of targeted literature searches for key studies, systematic reviews, and relevant articles were carried out to identify which causal or contextual factors related to IPV have the greatest scope for change. After conducting a wide range of research and reviewing literature using NVivo, with a particular focus on content and results from existing evidence-based interventions seeking to prevent IPV or address IPV risk factors, five causal factors and associated behaviours were selected (Table 3). A summary of each factor is provided below.

**Table 3:** Summary of factors that have greatest scope for change

6SQuID Step 2: Causal factors and behaviours with greatest scope for change
(1) Gender-equitable behaviours and attitudes
(2) Involved and equitable co-parenting
(3) Equally distributed workload
(4) Conflict resolution skills
(5) Effective communication

### Gender-equitable behaviours and attitudes

The Bandebereho intervention [63] includes sessions on: ‘Gender Equality’ (which encourages participants to consider how gender norms impact the relationships and lives of men and women), in addition to sessions on distributing household and care work more equally between men and women. Notably, findings from a study on Bandebereho by Doyle et al. [63] revealed a reduction in men’s dominance in decision-making in the household and greater levels of men’s participation in household tasks and childcare.

### Involved and equitable co-parenting

The Bandebereho programme [63] and the REAL Fathers intervention [14] include sessions on becoming a better parent by being an involved father, as well as sessions on becoming a more supportive partner by communicating effectively and being more involved in childcare and housework. For instance, Bandebereho includes sessions such as ‘Becoming a Father’, encouraging men to reflect on the benefits of being an engaged father and how to support both the mother and children [63]. Following their study, Doyle et al. [63] found an increase in men’s accompaniment to antenatal care and women in the intervention reported greater levels of partner support during pregnancy compared to the control group [63]. Similarly, REAL Fathers seeks to help participants develop effective couple communication and problem-solving skills [14]. Notably, findings from a study on REAL Fathers by Ashburn et al. [14] revealed both a reduction in IPV perpetration in addition to reductions in physical punishment of children.

### Equally distributed workload

Bandebereho also includes a session on ‘Sharing Responsibilities at Home’, encouraging participants to consider how gender roles impact division of work at home (e.g., childcare and household work) and how to distribute this more equally [63]. Findings from their study indicated greater levels of men’s participation in household tasks and childcare, improvements in division of housework, in addition to women in the intervention group reporting reductions in past-year physical and sexual IPV [63].

### Conflict resolution skills

MAISHA is a microfinance and gender training intervention seeking to reduce violence against women [64]. One of the ways the gender training component seeks to prevent IPV is through helping participants develop relationship skills such as non-violent, conflict resolution strategies. Findings from a cluster RCT of MAISHA indicated that at follow-up, compared to the control, women participating in the programme were less likely to experience physical IPV and express attitudes accepting of IPV [64]. Similarly, the Bandebereho intervention also includes content on conflict resolution strategies [63]. Results from their study showed that compared to the control, women in the intervention reported less past-year physical and sexual IPV. Men and women in the intervention group also reported less child physical punishment [63].

### Effective communication

REAL Fathers [14] also includes content that aims to build effective couple communication skills and promote collaborative problem-solving skills between partners. Findings from their intervention showed significant positive effects on couple communication. Men who participated in group mentoring and individual sessions had twice the odds, compared to those who did not, of the following: listening to their spouse, telling their spouse they appreciated them, and speaking with their partner about things that make them happy or frustrated, at both short- and longer-term follow-up [14].

### Step 3: Identify how to bring about change: mechanisms of change

Step 3 in the 6SQUID framework involves determining the specific mechanisms through which the factors identified in Step 2 might be able to change. More specifically, Step 3 focuses on the programme theory behind the intervention and how the programme seeks to bring about the intended outcome. A vital part of this is therefore the so-called change mechanism of the intervention. As such, underpinning an intervention with relevant theory in order to initiate change is key [38, 65]. Outlined below are the theories drawn upon when developing the IPV prevention content aimed at initiating the mechanisms of change. The mechanisms of change that underpin the IPV prevention content in the present study are the modifiable factors listed in column one of Table 4 and include: 1) Gender-equitable behaviours and attitudes, 2) Involved and equitable co-parenting, 3) Equally distributed workload, 4) Conflict resolution skills, and 5) Effective communication. These mechanisms of change are based on the shared risk factors identified in Step 2.

**Table 4:** Identified modifiable factors, IPV prevention topics, and example text-messages in ParentText

Modifiable Factors	IPV Prevention Topics	Example messages (version for fathers)
Gender-equitable behaviours and attitudes	(1) Treat each other as equals	“Family and friends might tell you how a husband or a father should act. But both men and women benefit when they talk to each other and make decisions together. For example, next time a decision needs to be made, involve your partner, and ask what they think!”
Involved and equitable co-parenting	(2) Become a confident parent and supportive spouse	“Get involved! When fathers are engaged in parenting their children, both the child, mother, and father benefit. Set aside some time today to spend with the children.”
Equally distributed workload	(3) Share family responsibilities	“Sharing family responsibilities with your partner can make life less stressful. Think of ways you can share the workload. Doing tasks together can also make them more fun.”
Conflict resolution skills	(4) Resolve conflict peacefully	“All adults have disagreements sometimes. But fighting is not an effective way to solve issues. Instead, if you start feeling angry, take a deep breath first and then respond in a calmer way.”
Effective communication	(5) Listen and talk to each other	“Listening and talking to those around us are key to a more peaceful home. Talking to your partner about issues before they become bigger problems can help avoid arguments from building up.”

### Programme theory

The Parenting for Lifelong Health (PLH) programmes, including ParentText, are primarily based on social learning theory and attachment theory, and consist of content based around themes of relationship building, positive reinforcement, limit setting, and effective discipline [66]. The digital adaptations of the in-person PLH programmes, including ParentText, are also grounded in these theoretical approaches. In ParentText, the IPV prevention content also draws upon gender-transformative approaches used in interventions been found to reduce IPV and improve gender-equitable attitudes (see Step 1 and 2). Gender transformative interventions are those which are **‘**designed specifically to encourage men and boys to adopt and enact gender-equitable, nonviolent attitudes and behaviours’ ([67]: p.1635–1636), and often seek to target harmful social and gender norms – a key shared risk factor identified and discussed in Step 1. This approach has seen a rapid increase in recent years, and in a systematic review on gender-transformative interventions by Casey et al. [68], six out of the 10 interventions found a statistically significant effect on one or more outcomes related to IPV, gender-equitable attitudes and behaviours.

### Theory of change

**Fig. 1:**
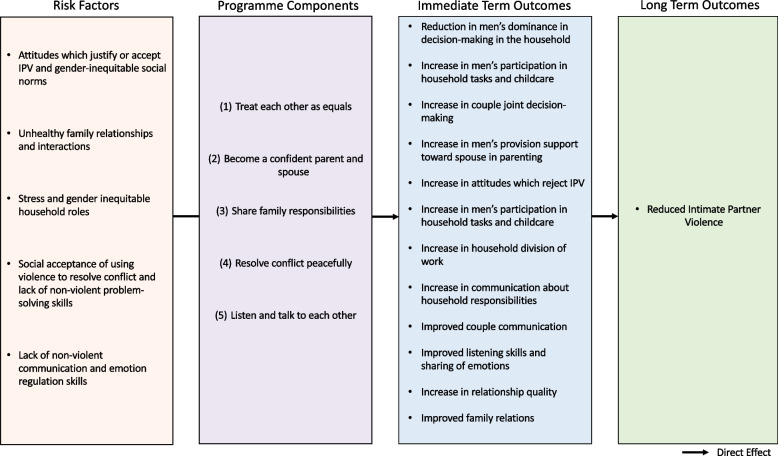
Theory of Change: ParentText IPV Prevention Content

 The content in ParentText draws upon material from various evidence-based interventions found to improve gender-equitable attitudes and reduce IPV (elaborated in Step 2) [14, 63, 64]. Using the risk factors identified in Step 2, and following preliminary consultations with the selected group of academic researchers (*n* = 5) and practitioners (*n* = 5), through virtual interviews and written feedback provided via email, five topics of focus were selected for the ParentText IPV prevention content: 1) Treat each other as equals; 2) Become a confident parent and supportive spouse; 3) Share family responsibilities; 4) Resolve conflict peacefully; 5) Listen and talk to each other. The proposed causal pathway between the identified risk factors, the IPV prevention topics (programme components), and behaviour change domains, are illustrated in the theory of change in Fig. 1.

### Programme: IPV prevention content

We then created specific intervention content related to each modifiable factor identified in Step 2. Using the findings from Step 1 and 2, we drafted a set of core text-messages to accompany each topic (Table 4). Given that research findings indicate the importance of addressing multiple risk factors simultaneously when seeking to reduce IPV [69], the IPV prevention topics and text messages created sought to target various of the risk factors identified (as demonstrated in Table 4).

These text-messages were tested with grassroots organisation partners (*n* = 7), including but not limited to, Parenting Partners Caribbean (PPC), UNICEF Jamaica, Clowns Without Borders South Africa, and the Jamaica National Parenting Support Commission (NPSC), in virtual consultation sessions where feedback was provided to ensure the text-messages and content was appropriate for the target audience. The material was also reviewed by academic researchers (*n* = 5) and practitioners (*n* = 5), who provided written and verbal input via email and in individual consultation meetings (see Additional file 5. Consultation focal points for full list of stakeholders). The intervention content is described in accordance with the Template for Intervention Description and Replication (TIDieR) checklist (Additional file 1) and the guideline for reporting intervention development studies (GUIDED) (Additional file 2). Further details are provided in accordance with TIDieR in Additional file 3.

### Check-in messages and multimedia content

**Fig. 2:**
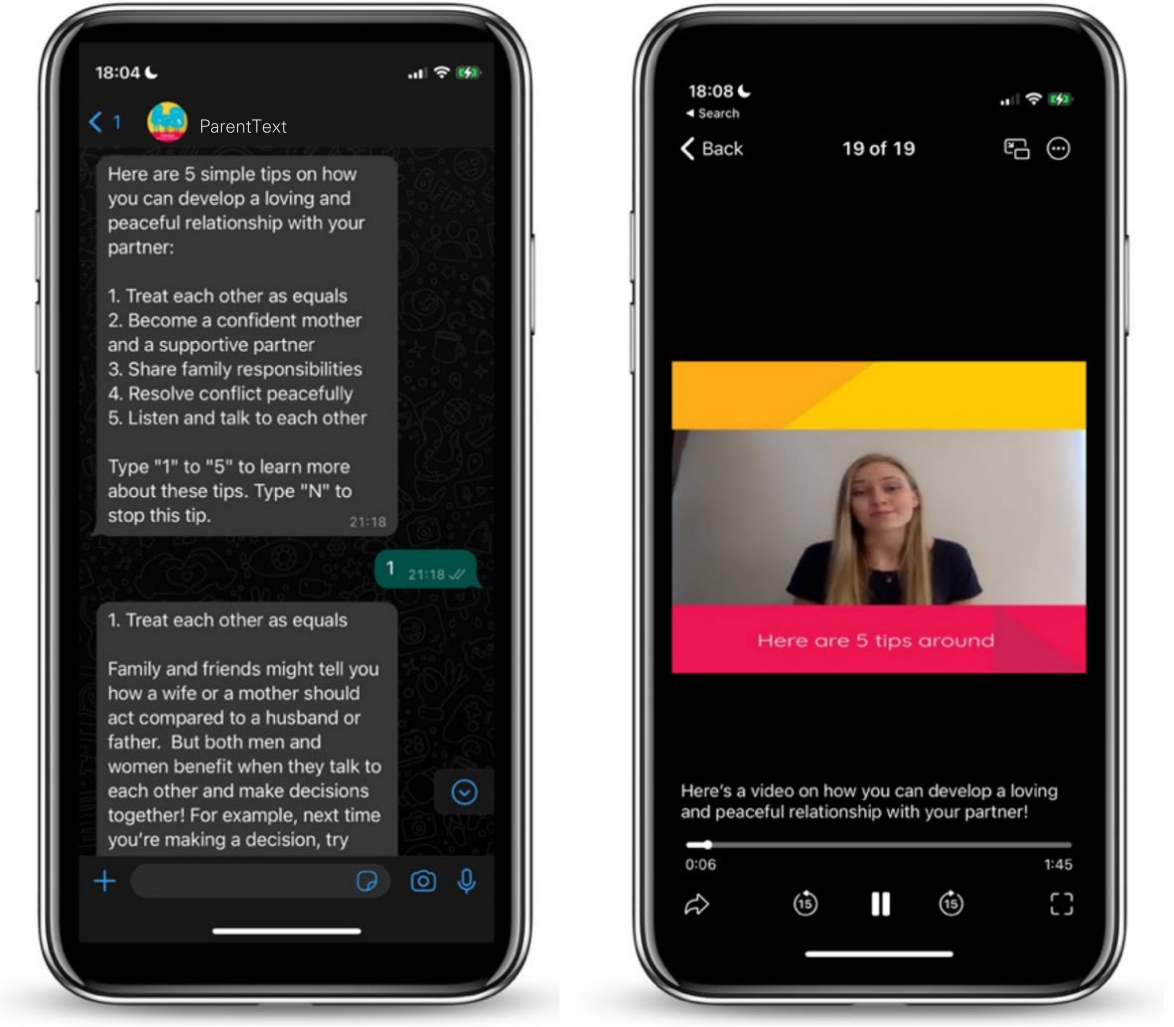
Visual demonstration of the IPV prevention content delivered via text and video in ParentText

**Fig. 3:**
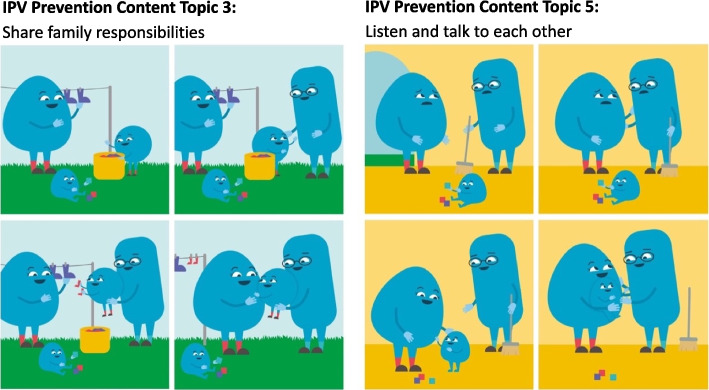
Example of illustrations of IPV prevention material integrated in ParentText

We also developed check-in messages and multimedia material (Fig. 2). The check-in messages seek to remind parents of activities and provide troubleshooting support. One check-in message for each IPV prevention topic is delivered every two days following the IPV material (see Additional file 6 for examples). A variety of multimedia content, including cartoons and videos, was also created (Fig. 3).

### Step 4: Identify how to deliver the change mechanism

While Step 3 involves identifying *what* the change mechanism is, Step 4 focuses on determining how to *deliver* the change mechanism [38]. Hence, for Step 4, a programme schedule was designed (see Table 5). (For the full 37-day ParentText schedule see Additional file 4). Once developed, the programme schedule underwent further revisions after being reviewed by the partner grassroots organisations (*n* = 7) and researcher stakeholder group (*n* = 5), who provided their perceptions on the programme structure.

**Table 5:** Structure of IPV prevention content and check-in message schedule in ParentText

Day	IPV Prevention Content
Day 1	Programme starts
Day 3	IPV Baseline assessment
Day 8	**Main IPV Prevention Content:** IPV Prevention Content Topics 1–5
Day 11	**Check-in**: IPV Topic 1 (Treat each other as equals)
Day 14	**Check-in**: IPV Topic 2 (Become a confident parent and supportive spouse)
Day 18	**Check-in:** IPV Topic 3 (Share family responsibilities)
Day 22	**Check-in:** IPV Topic 4 (Resolve conflict peacefully)
Day 26	**Check-in:** IPV Topic 5 (Listen and talk to each other)
Day 37	Programme ends

### Step 5: Test and refine on a small scale: formative evaluation in Jamaica

#### Formative evaluation: methods

##### Setting and population

The formative evaluation study was carried out in Kingston, Jamaica from June to December 2022. Inclusion criteria for participants in this formative evaluation study were parents and caregivers over 16 years of age with no upper age limit, currently caring for a child between the ages of 0 to 17 years, currently in a partnered relationship, had access to a phone that could receive messages via WhatsApp, and provided consent to participate in the study. Exclusion criteria included participants who were not caregivers, parents, or currently caring for a child and participants who were not currently in a partnered relationship. Participants were recruited via a recruitment message sent out in collaboration with UNICEF Jamaica, the Spotlight Initiative, and U-Report, a social messaging tool pioneered by UNICEF available on multiple messaging, SMS, and social media channels [70, 71]. The recruitment message included a link to the intervention embedded within a WhatsApp, Facebook Messenger, or Telegram business account. Participants that clicked on the link were directed to the account and were asked if they consented to participate in the intervention and evaluation with a link to a more detailed information sheet. No compensation was provided to participants for participation.

##### Data collection

In total, 96 partnered participants took part in the evaluation study. Participants who indicated that they were either married or partnered were invited to respond to IPV assessments on a secure external server, via Oxford’s Linux virtual machine using Online Data Kit (ODK) to ensure that no responses were saved on their mobile devices. All personal identifying data were deleted as soon as end line data collection was completed. Further ethical and safety considerations are elaborated on in the ethics section. The IPV assessments were optional, and parents could refuse to answer them but still receive parenting tips. Before they were invited to respond to the IPV assessment, participants were asked for permission and were reminded that their answers would be completely private. The IPV assessments were delivered at baseline and at 1-month post-intervention. Engagement data, including engagement rate (days active in program) and interaction with the IPV content was also examined to gain a better understanding into the participants’ experience of interacting with the chatbot.

##### Data analysis

Descriptive statistics were used to examine participants’ level of engagement with the chatbot. We also explored interactions with the IPV content by using descriptive statistics to examine participants’ behaviour and engagement related to the IPV material, topics, and check-ins provided in the intervention.

##### Ethics

Participants were screened at the beginning of the IPV assessment to ensure that men and women from the same household were not both interviewed about IPV. This is based on WHO’s violence against women (VAW) research guidelines [72] and ethical and safety recommendations for intervention research on VAW to minimise any risk of harm [73]. Accordingly, men who reported that their partner was participating in the programme were not asked the IPV questions from the IPV assessment and were instead only asked questions on gender-equitable behaviours and on attitudes toward IPV and gender roles as part of the embedded surveys. Participants who reported IPV experiences were automatically provided with referrals to services supporting individuals experiencing violence customised to their local context. Participants were also able to access local referral details by writing “Help Me” and selecting “Other Support” in the ParentText free text field. ParentText is also designed to identify high-risk keywords to detect potential disclosure of dangerous situations in the free text field. After detection, ParentText is automated to provide the participant with an empathetic and empowering response with referral contact details that are localised to the country that supports parents and child safety (e.g., police, ambulance, hotlines).

### Formative evaluation: results

#### Participants characteristics

Participants ranged in age from 16 to 51 years old (M = 33.48; SD = 8.09). Most participants were female (85%) and 5% were male (see Table 6 for a detailed breakdown). Forty-three percent of caregivers reported their relationship status as married, and 53% as partnered.

**Table 6:** Characteristics of sample

Sociodemographic characteristics	Participants (*N* = 96)
Age	33.5 years
*Gender*	
Female	85 (88%)
Male	4 (5%)
Non-binary	3 (3%)
Not stated	4 (4%)
*Relationship status*	
Married	43 (44%)
Partnered	53 (55%)

Data are N (%) or mean (standard deviation)

#### User engagement outcomes

Overall, the average engagement rate was 0.50 interactions per day, in other words, interacting with the intervention once every other day (Table 7). The average length in the programme was 14 days (SD = 56.42). In terms of engagement, when prompted to view the relationship material introduction, approximately half of participants (51%) responded “yes” and 49% responded “no” or didn’t respond. When provided with the option to view additional content on the topic of relationships, 45% responded “yes”; 55% didn’t respond; only 3% responded “no”, and 1% did not select any of these options and instead responded by typing a question about parenting as a stepfather in the textbox. Across the five relationship topics, *Relationship Topic 1* (‘Treat each other as equals’) was viewed by the largest proportion of participants (28%), as well as revisited and viewed the greatest number of times in total (34 times). Following this, *Relationship Topic 2* was viewed by 25% of participants, *Topic 3* by 21% of participants, *Topic 4* by 20% of participants, and *Topic 5* by 22% of participants. A total of 15% of all participants selected to view all five relationship topics.

**Table 7:** ParentText user engagement outcomes

**Overall engagement**		
Engagement rate (interactions per day)	0.50 interactions per day	
Average length in programme	14 days (56.42)	
**IPV content engagement**		
**Selected to view the relationship material introduction**	Total of sample (%)	
Responded “Yes”	49 (51%)	
Responded “No” / Didn’t respond	47 (49%)	
**Selected to view the additional topics**		
Responded “Yes”	41 (43%)	
Responded “No”	3 (3%)	
Didn’t respond	51 (53%)	
Other response	1 (1%)	
**Engagement with relationship topics**	**Participants who viewed content (% of total sample)**	**Total number of times each topic was viewed**^**a**^
**Relationship Topic 1** ‘Treat each other as equals’	27 (28%)	34
**Relationship Topic 2** ‘Become a confident parent and supportive spouse’	24 (25%)	28
**Relationship Topic 3** ‘Share family responsibilities’	20 (21%)	24
**Relationship Topic 4** ‘Resolve conflict peacefully’	19 (20%)	21
**Relationship Topic 5** ‘Listen and talk to each other’	21 (22%)	28
**All Relationship Topics** (Participants who viewed all topics)	14 (15%)	–

^a^Some participants viewed each topic more than once as they had the opportunity to revisit the material after each Check In message

In total, the ParentText programme lasted 37 days, with the IPV Relationship material delivered in the first 26 days. Overall, 23% of participants stayed in the programme long enough to receive the *Relationship Check In 1* delivered on Day 11 of the programme (Table 8). Notably, by Day 14, only 13% of participants were in the programme to receive *Relationship Check In 2.* By Day 18, when *Relationship Check In 3* was delivered, 6% of participants remained in the programme*.* Notably, 3% of participants remained by Day 22 and 1% by Day 26 when *Check In 4* and *Check In 5* were delivered respectively.

**Table 8:** Stayed in programme long enough to receive IPV Check In messages

**Participant retention**	Total of sample (%)^a^ who received relationship Check In messages
**Relationship Check In 1** (day 11) ‘Treat each other as equals’	22 (23%)
**Relationship Check In 2** (day 14) ‘Become a confident parent and supportive spouse’	13 (13%)
**Relationship Check In 3** (day 18) ‘Share family responsibilities’	6 (6%)
**Relationship Check In 4** (day 22) ‘Listen and talk to each other’	3 (3%)
**Relationship Check In 5** (day 26) ‘Listen and talk to each other’	1 (1%)

^a^Out of the total sample who received IPV prevention content (96 participants)

## Discussion

This article describes and examines the development, creation, and evaluation of the IPV prevention content produced for the digital parenting intervention ParentText. The design of the intervention content followed the steps in the 6SQuiD intervention development model [38], which builds on the MRC UK framework for developing and evaluating complex health interventions [39]. This process involved conducting targeted literature searches and reviewing existing interventions to guide the selection of the intervention content and theory used, as well as consulting researchers, practitioners, and members of grassroots organisations throughout for input and feedback on the intervention content and design. A formative evaluation was also conducted to gain preliminary insights on user engagement with the chatbot in order to identify areas with room for further improvement and intervention refinement.

To our knowledge, this study is the first to report the development process of integrating IPV prevention content into a digital parenting intervention. It offers a unique and important contribution both to the field of violence prevention as well as to research on digital interventions. Indeed, while there is growing evidence surrounding the use of technology-based and digital programmes in public health, there is a paucity of research on digital interventions for IPV prevention, in particular, in addressing multiple forms of family violence [74]. Notably, even though there exist some technology-based studies addressing family violence, most focus on the secondary and tertiary prevention of IPV (i.e. response interventions), rather than on primary prevention [74, 75]. As such, the present study offers an important first step in examining the development and preliminary evaluation of integrating primary IPV prevention strategies into a parenting chatbot. The formative evaluation provides valuable insights on the importance of examining and identifying strategies to enhance user-engagement outcomes in digital interventions. The retention rates in the formative evaluation (a median 11-day retention of 23% and a median 22-day retention of 3%) were similar to that of other digital intervention studies. For instance, a study by Baumel et al. [76], examining user engagement with digital interventions and apps focusing on mental health, found that overall user retention rates were low, with median 15-day and 30-day retention rates consisting of 3.9% and 3.3% respectively. Whilst low retention rates in smartphone-delivered interventions are not uncommon and have been noted as one of the main challenges in the field [77], numerous studies have revealed potential solutions and strategies that may increase retention and enhance user engagement. For example, a meta-analysis on attrition rates in smartphone-based interventions by Linardon and Fuller-Tyszkiewicz [78] found that providing incentives and reminding participants to engage in the programme, significantly reduced attrition rates.

Similarly, numerous studies have also found for digital interventions with optional or unguided components, it is not uncommon for users to exit the intervention before engaging with all of the programme material [76]. These findings were also noted in our present study. For instance, in our formative evaluation, 45% of participants selected “Yes” when presented with the option to view additional relationship topics in ParentText, however, only 15% of participants viewed all five of the relationship topics. Interestingly, these results are very similar to findings reported in other studies. A systematic review of user engagement in mental health digital interventions, for example, revealed that rates of completion of all programme modules in the interventions ranged from 2.8% to 19.5% [79]. These metrics are comparable to another study where researchers found that 17% of participants in the intervention selected to view the supplemental material provided [80]. Despite these challenges, digital interventions also offer unique opportunities in terms of providing scalable, low-cost, and accessible solutions for disseminating interventions [81]. Combined with promising research findings that have revealed how digital interventions and text-messaging based programmes have been used successfully to deliver critical behaviour change techniques and enhance access to local health resources [82, 83], exploring ways to improve user engagement with digital programmes remains critical.

Overall, the results from the formative evaluation suggest there is room for refinement in terms of improving user engagement and the retention rate in the programme. Given that the evaluation study revealed that only 23% of participants remained in the programme by day 11 (out of 37 days), this highlights that the way the content is delivered, and the programme length overall, may need to be adjusted in future iterations of the programme. This is reiterated by the average length in the programme which was a median of 14 days. In the field of parenting interventions, findings from numerous meta-analyses suggest that shorter interventions produce greater positive change, both in technology-based parenting interventions [84] and in-person programmes [85].These findings are being incorporated in the next version of the programme that is currently being tested in a pre-post study, where the programme length has been cut by almost 50%, from 37 to 23 days. A qualitative research study is also currently being planned with participants in order to better improve the design and content of the intervention to enhance user engagement and retention. Furthermore, given that the findings from the evaluation study revealed that 20–28% of participants selected to view the relationship topics, in future studies it would be important to gain further insights and explore more strategies, such as goal setting, to increase user engagement with the programme content.

### Strengths and limitations

There are various strengths of this intervention content development and formative evaluation study. The evidence- and theory-informed, stepwise approach used for the intervention content development is a major strength of the present research. Providing a transparent and detailed documentation of the content development process, allows for intervention content to be appraised, replicated, and improved [86]. The specific content in the programme the participants received is also personalised based on each user, with options to use a low-data mode for users who prefer text-only format and differences in the language used for fathers and mothers to make the content more personalised, which has been suggested to increase intervention efficacy in previous text messaging-based interventions [30].

There are also a few limitations worth addressing. Difficulties surrounding access to internet or credit for mobile data remains a major challenge with digital interventions. Similarly, technological problems, such as lack of memory space on digital devices is also a common issue when using digital resources [87]. Whilst the programme included content personalised for male caregivers, recruitment of men still appeared to be a challenge in the present study, with only 5% of participants identifying as male. While the low representation of male caregivers in the present study is a common challenge in the parenting programme field [13], this underscores the importance of making further adjustments to programme content and recruitment strategies to ensure male caregivers are also included in future studies. In addition, the present paper, while including a formative evaluation, due to the lack of a control group, was not able to examine the effectiveness of the programme content on IPV outcomes. As such, future studies would benefit from using more rigorous methods to optimise and evaluate the programme, both in terms of effectiveness as well as by investigating what the ‘active’ ingredients of change are in the intervention [88].

## Conclusion

The COVID-19 pandemic has contributed to a substantial increase in rates of IPV as well as violence against children worldwide [4]. Given that rates of IPV were already extremely high pre-pandemic, there is now an even greater need for evidence-based prevention efforts and scalable interventions that address this form of violence. Alongside a growing awareness surrounding the intersections that are shared by IPV and other forms of violence in the home, such as violence against children, parenting programmes are uniquely positioned to target both forms of violence concurrently. With a rise of digital interventions in the public health sphere, digital parenting programmes offer a valuable opportunity to target both intergenerational forms of violence as well as in terms of addressing key risk factors of IPV. The IPV prevention content that was empirically and iteratively developed, as outlined in this paper, offers a clear example of how IPV prevention material can be integrated into a digital parenting intervention. Whilst further research is necessary to establish intervention effectiveness, this article and the results from formative evaluation have provided valuable preliminary insights surrounding patterns in user engagement in the intervention and the programme content, shedding light on elements of the chatbot where there is room for refinement to enhance engagement.

### Supplementary Information

**Additional file 1.** TIDieR Checklist.

**Additional file 2.** GUIDED.

**Additional file 3.** ParentText TIDieR Description.

**Additional file 4.** Intervention Schedule.

**Additional file 5.** Focal Points.

**Additional file 6.** Example Messages.

## Data Availability

The datasets used and analysed during the current study are available from the corresponding author on reasonable request.

## Abbreviations

6SQuID: Six Steps in Quality Intervention Development
GUIDED: Guideline for reporting intervention development studies
IPV: Intimate partner violence
LMIC: Low- and middle-income country
NPSC: National Parenting Support Commission Jamaica
PLH: Parenting for Lifelong Health
PPC: Parenting Partners Caribbean
REAL: Responsible Engaged, and Loving Fathers Initiative
TIDieR: Template for Intervention Description and Replication
UN: United Nations
UNICEF: United Nations Children’s Fund
VAC: Violence against children
VAW: Violence against Women
VAWG: Violence Against Women and Girls
WHO: World Health Organization
