# Supplementary material for: Integrating intimate partner violence prevention content into a digital parenting chatbot intervention during COVID-19: Intervention development and remote data collection
Source: BMC Public Health. 2023 Sep 4;23:1708. doi: 10.1186/s12889-023-16649-w (PMC10476288; doi:10.1186/s12889-023-16649-w)
Supplement: Supplementary file 3 — Additional file 3. ParentText TIDieR Description. [file 12889_2023_16649_MOESM3_ESM.pdf]

Additional file 3: Details of ParentText and the integrated intimate partner violence prevention content described according to TIDieR

Item		Description
1.	Brief name	ParentText: Intimate Partner Violence (IPV) Prevention Content
2.	Why	In recent years, a number of shared risk factors have been identified between violence against children and intimate partner violence, with a particular emphasis harmful gender and social norms that condone violence. Accordingly, calls for an increase in violence prevention efforts that target both forms of violence concurrently have been growing. The gender-transformative and partner relationship content in ParentText thus seeks to address these risk factors associated with harmful gender and social norms, in order to prevent and reduce IPV and violence in the family. The underlying theories of the ParentText programme and of the IPV prevention content are Social Learning Theory (SLT), Attachment Theory, and gender-transformative approaches which champion the adoption and enactment of gender-equitable, nonviolent attitudes, and behaviours to prevent gender-based violence and harm.
3.	What (Materials)	The content material developed draws upon existing evidence-based violence prevention interventions including the Bandebereho programme (1)and the REAL Fathers intervention (2), the MAISHA microfinance and gender training intervention (3).
4.	What (Procedures)	The intervention content in ParentText seeks to increase parents' and caregivers' understanding of the effect of harmful gender stereotypes and attitudes, as well as provide them with examples and skills on how to they can become a more supportive parent and partner. This material is delivered both in the form of text-based messages as well as via multi-media content such as through cartoons and videos. The programme also uses 'check-in' messages to follow-up on the material introduced earlier in the intervention and asks users at regular intervals how they are progressing with practicing the different skills they have learnt. This allows users to revisit the material and provides them with gentle reminders and examples of how they can further incorporate more gender-equitable attitudes and behaviours both as a partner and parent.
5.	Who (Provides)	The intervention is delivered through RapidPro, an open-source application that serves low-income communities.
6.	When and how much (Mode of delivery)	The material is delivered via text-messages and multi-media content through messaging platforms such as Telegram, WhatsApp and Facebook messages, and via SMS for individuals without smartphone access. Participants receive messages over 5 to 12 weeks, depending on the frequency of delivery.

9.	Tailoring	The content is personalised based on the gender of the participant using the programme and based on the data availability of the mobile device (with options for text-only or a combination of text, images, and video). ParentText content has also been adapted with implementing partners in multiple LMICs to ensure that the content is culturally relevant and available in multiple local languages.
10.	Modifications	Future iterations will be made shorter. Revisions for future iterations are discussed in the Discussions section of the paper.
11.	How well (planned)	Fidelity to the program was assessed based on engagement data (see Results section of the paper) and via qualitative interviews and focus group discussions (the latter of which are covered in subsequent publications).
12.	How well (actual)	Engagement data assessing the actual adherence is reported in the present paper, and further information on adherence based on findings from qualitative interviews and focus group discussions with users will be covered in subsequent publications.

Note: TIDieR: Template for Intervention Description and Replication (4)
