# Supplementary material for: Integrating intimate partner violence prevention content into a digital parenting chatbot intervention during COVID-19: Intervention development and remote data collection
Source: BMC Public Health. 2023 Sep 4;23:1708. doi: 10.1186/s12889-023-16649-w (PMC10476288; doi:10.1186/s12889-023-16649-w)
Supplement: Supplementary file 4 — Additional file 4. Intervention Schedule. [file 12889_2023_16649_MOESM4_ESM.pdf]

Additional file 4: Structure of the 37-day version of the ParentText Intervention

Day	Morning	Main	Evening
1		Welcome	
2	Content: One-on-one time	Supportive: Activities	Supportive: Share
3	**IPV Baseline assessment**	Content: Take a pause	Supportive: Praise/Calm
4	Supportive: Sharing	Content: Positive introduction	Supportive: Praise/Calm
5	Supportive: Praise/Calm	Content: Positive instructions	Check-in: One-on-one time
6	Content: Quick Pause	Content: Book sharing	Supportive: Praise/Calm
7	Check-in: Connection	Content: Routines	Supportive: Praise/Calm
8	Check-in: Instructions	**Content: IPV Main Material**	Supportive: Praise/Calm
9	Supportive: Praise/Calm	Supportive: Praise/Calm	Check-in: Book sharing
10	Supportive: Praise/Calm		Supportive: Praise/Calm
11	Check-in: Routine	Content: Positive rules	**Check-in: IPV Topic 1 - Treat each other as equals**
12	Supportive: Praise/Calm	Supportive: Help reminder	Supportive: Praise/Calm
13	Check-in: Covid	Content: Education	Supportive: Share
14	Supportive: Praise/Calm	Supportive: Disabilities	**Check-in: IPV Topic 2 - Become a confident parent and supportive spouse**
15	Supportive: Praise/Calm	Content: Online	Content: Relax
16	Supportive: Praise/Calm	Supportive: Help reminder	Supportive: Praise/Calm
17	Check-in: Rules	Content: Redirection	Content: Anger management
18	Supportive: Praise/Calm	Supportive: Sharing	**Check-in: IPV Topic 3 - Share family responsibilities**
19	Supportive: Praise/Calm	Content: Behaviour / Crying	Check-in: Education
20	Supportive: Praise/Calm	Supportive: Activities	Supportive: Praise/Calm
21	Check-in: Online	Content: Consequences	Supportive: Activities
22	Supportive: Praise/Calm	Supportive: Help reminder	**Check-in: IPV Topic 4 - Resolve conflict peacefully**
23	Supportive: Praise/Calm	Content: Problem solving	Supportive: Praise/Calm
24	Supportive: Praise/Calm	Supportive: Behaviour	Supportive: Praise/Calm
25	Check-in: Redirect	Content: Emotion	Supportive: Praise/Calm
26	Supportive: Praise/Calm	Supportive: Help reminder	**Check-in: IPV Topic 5 - Listen and talk to each other**
27	Check-in: Crying	Content: Behaviour / Crisis	Supportive: Praise/Calm
28	Supportive: Praise/Calm	Supportive: Sharing	Supportive: Praise/Calm
29	Supportive: Praise/Calm	Content: Budgeting	Check-in: Consequences
30	Supportive: Praise/Calm	Supportive: Activities	Supportive: Praise/Calm
31	Supportive: Praise/Calm	Content: Loving Kindness	Check-in: Problem solving
32	Supportive: Praise/Calm	Supportive: Reminder	Supportive: Praise/Calm
33	Check-in: Emotions	Content: Exercise	Supportive: Praise/Calm
34	Supportive: Praise/Calm	Supportive: Reminder	Supportive: Praise/Calm
35	Supportive: Praise/Calm	Content: Anger management	Check-in: Behaviour / crisis
36	Supportive: Praise/Calm	Supportive: Help reminder	Supportive: Praise/Calm
37	Celebrate finishing programme		

Note: IPV prevention content is noted in bold font. Types of messages include in the programme include Content messages, Check-in messages and Supportive messages.
