# Supplementary material for: Integrating intimate partner violence prevention content into a digital parenting chatbot intervention during COVID-19: Intervention development and remote data collection
Source: BMC Public Health. 2023 Sep 4;23:1708. doi: 10.1186/s12889-023-16649-w (PMC10476288; doi:10.1186/s12889-023-16649-w)
Supplement: Supplementary file 5 — Additional file 5. Focal Points. [file 12889_2023_16649_MOESM5_ESM.pdf]

Additional file 5: Consultation focal points

Academic researchers
Alessandra Guedes – UNICEF Innocenti, Italy<br>Dr Heidi Stoeckl – Ludwig Maximilian University of Munich, Germany<br>Professor Dr Rumaya Binti Juhari – Department of Human Development & Family Studies, Faculty of Human Ecology, University Putra Malaysia<br>Dr Rosanne M. Jocson – Ateneo de Manila University, the Philippines<br>Dr Joyce Wamoyi – Tanzania National Institute of Medical Research, Tanzania
Violence prevention professionals and stakeholders
Helena Duch – Oak Foundation Head of Solutions Sub-Programme (Prevent Child Sexual Abuse Programme), Switzerland<br>Blain Teketel – Oak Foundation Programme Officer, Child Abuse Programme, East Africa, Ethiopia<br>Ytske Van Winden – International Child Protection Consultant, Gender Unit, UNICEF Jamaica<br>Ross Sheil – Communications Officer, Digital Media, UNICEF Jamaica<br>Elizabeth Dartnall – Executive Director, Sexual Violence Research Initiative (SVRI)
Local community organisations and institutions
Mpume Danisa and colleagues – Clowns Without Borders South Africa (CWBSA)<br>Susie Mjwara – Clowns Without Borders South Africa (CWBSA)<br>Jamaica National Parenting Support Commission (NPSC)<br>Colleen Wint-Bond and colleagues – Parenting Partners Caribbean (PPC)<br>Janet Brown – Parenting Partners Caribbean (PPC)<br>Glynis Salmon – Parenting Partners Caribbean (PPC)<br>Linda Craige-Brown – Parenting Partners Caribbean (PPC)
