# Supplementary material for: Integrating intimate partner violence prevention content into a digital parenting chatbot intervention during COVID-19: Intervention development and remote data collection
Source: BMC Public Health. 2023 Sep 4;23:1708. doi: 10.1186/s12889-023-16649-w (PMC10476288; doi:10.1186/s12889-023-16649-w)
Supplement: Supplementary file 6 — Additional file 6. Example Messages. [file 12889_2023_16649_MOESM6_ESM.pdf]

Additional file 6: Example of check-in message for Topic 1 ‘Treat each other as equals’

Check In 1

Hi! Have you tried involving your partner when making decisions recently? [**Yes / No]**

Well done! Communicating and working together with your partner makes a difference!
How was your experience?

1. Great	2. Neutral	3. Bad

[If they reply “Great”:]
That is fantastic! Would you like to review the content on this? (Yes / No)
That is okay! Remember you can access this content at any time by typing "Help". Chat to you later!

[If they reply “Neutral / Bad”:]
I am sorry to hear this. What did you find difficult about involving your partner when making a decision? Please select a number 1-5 from the following options:

1. I didn’t have time to involve my partner
2. I didn’t know how to involve my partner
3. I felt uncomfortable asking for my partner's opinion
4. I forgot to involve my partner
5. My partner reacted negatively to me when I tried

[Advice responses based on responses 1-5 above]:

**[Response 1]:**	**[Response 2]:**	**[Response 3]:**	**[Response 4]:**	**[Response 5]:**
It can sometimes be difficult to find the time to ask our partners for their opinions when making decisions. Think of a time during the day when both you and your partner might be less busy, such as early in the morning or later in the evening.	It can sometimes be hard to know how to involve your partner when making a decision. Sometimes all you need to do is to ask them what they think. This shows them you care and want them to be involved!	It can take time to get used to making decisions together. Give yourself time and keep trying. Remember that by communicating with your partner and involving them in decision-making, you will both eventually start to feel more comfortable doing so.	It can be hard to remember to involve our partners in decision making, especially when we are used to doing it alone. If you forget, it’s important to keep trying. Asking for their opinion shows that you care and want them to be involved.	Sorry to hear that your partner reacted negatively. Be proud of yourself for trying and be gentle with yourself. Remember you can always get extra support by typing "Help".
